# Bullous Erythema Elevatum Diutinum: A Case Report

**DOI:** 10.7759/cureus.90878

**Published:** 2025-08-24

**Authors:** Qian Lu, Cunwei Cao, Jiarong Liang, Jiaguang Su

**Affiliations:** 1 Department of Dermatology, The First Affiliated Hospital of Guangxi Medical University, Nanning, CHN

**Keywords:** erythema elevatum diutinum, histopathological examination, leukocytoclastic vasculitis, nodules, vesiculobullous variant

## Abstract

Erythema elevatum diutinum (EED) is an uncommon, chronic form of cutaneous leukocytoclastic vasculitis that typically manifests as purplish-red to reddish-brown nodules, papules, or plaques, predominantly on the extensor surfaces. We report a rare case of bullous EED in a 67-year-old Chinese woman, whose condition included an unusual blistering presentation. The diagnosis was confirmed through histopathological examination. Upon diagnosis, the patient was treated with a combination of oral prednisone, thalidomide, and local infiltration therapy with triamcinolone acetonide, which led to significant clinical improvement. This case is noteworthy for its atypical presentation and its broader implications for the diagnosis and management of similar cases.

## Introduction

Erythema elevatum diutinum (EED) is a rare, chronic form of leukocytoclastic vasculitis with an unclear etiology. Clinical manifestations are highly variable. While some patients may present with minimal skin lesions, others exhibit pruritic nodules and plaques, often symmetrically distributed. Typical lesions are persistent and consist of multiple papules, plaques, or nodules that range from reddish-brown to purplish-red and are elevated above the skin. These lesions are predominantly located on the limbs and the extensor surfaces of joints, particularly on the elbows, knees, ankles, hands, and fingers [1]. The vesiculobullous variant is a rare manifestation of EED, typically observed in HIV-positive individuals, with dapsone resistance being a common issue [2,3]. We report a rare case of the bullous variant of EED, which is characterized by vesiculobullous formation.

## Case presentation

**Figure 1:**
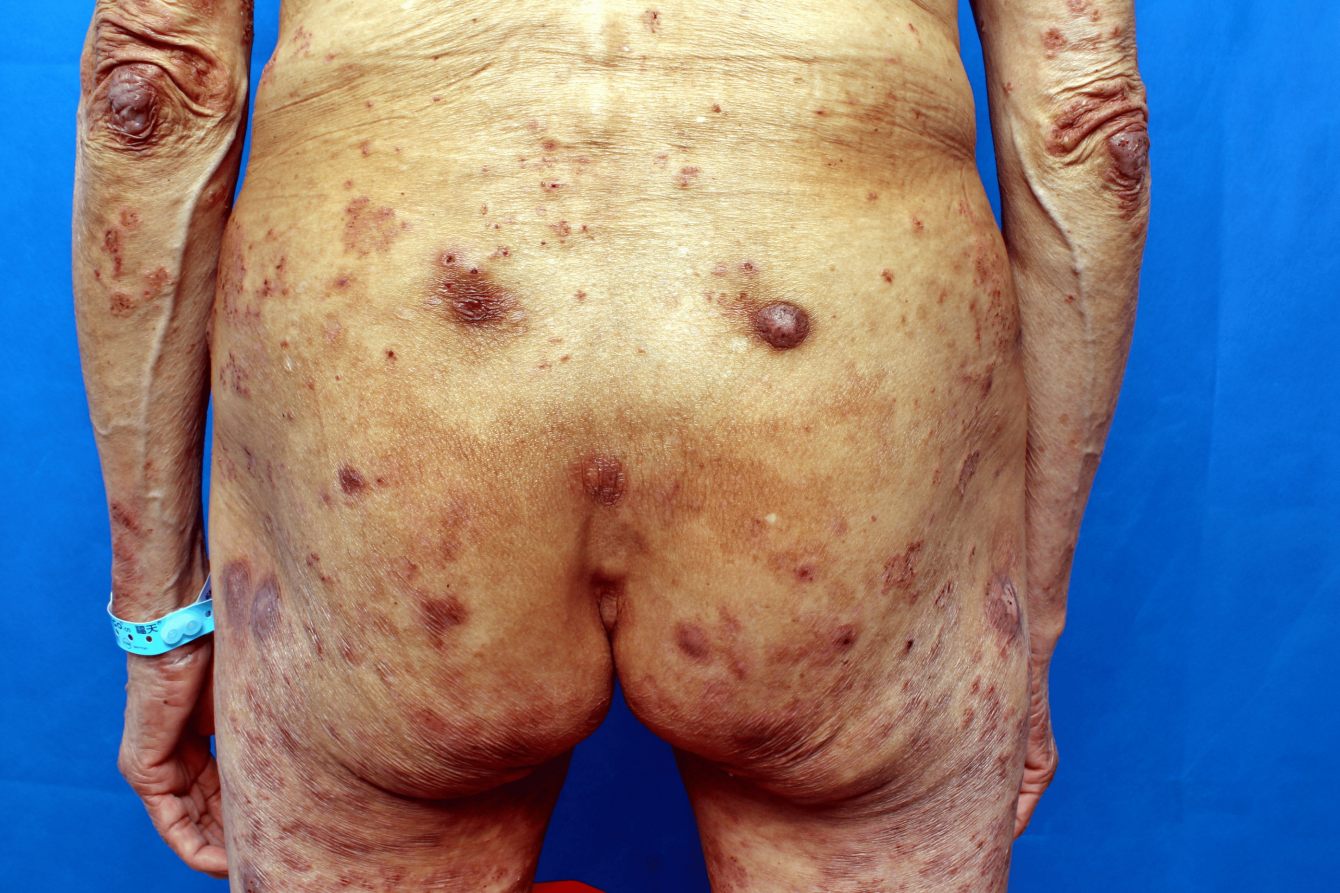
Erythema elevatum diutinum presenting as purplish-red plaques and nodules on the elbows and buttocks

**Figure 2:**
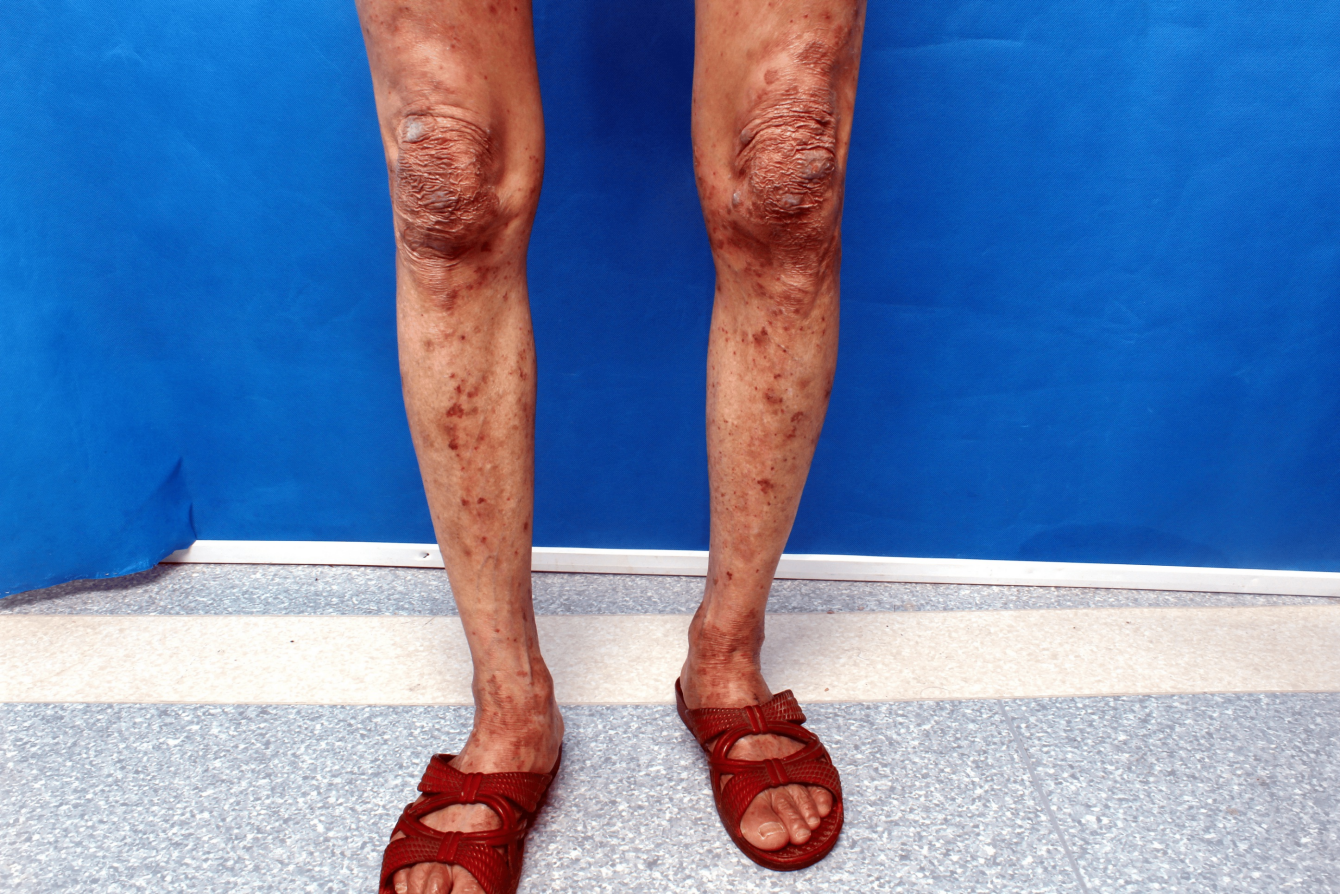
Clinical image showing purplish-red plaques and nodules on the knees

A 67-year-old Chinese woman presented with a five-year history of progressive cutaneous lesions, including erythema, papules, vesicles, and nodules, accompanied by pruritus. These lesions affected her extremities and gluteal region. The initial symptoms began five years ago with the spontaneous appearance of erythematous patches and papules on her extremities and buttocks, without identifiable precipitating factors. Initially diagnosed as eczema, her condition showed transient improvement with antihistamines and topical corticosteroids, but relapsed upon discontinuation of treatment, eventually progressing to vesicular eruptions. Over time, the condition developed violaceous plaques and nodules, predominantly located on her knees, elbows, calcaneal regions, and buttocks (Figures 1-2).

The patient denied experiencing any fevers, chills, cardiopulmonary symptoms, abdominal pain, diarrhea, oral ulcers, joint pain, or unintentional weight loss. Her medical history was notable for bronchial asthma, and both her personal and family histories were unremarkable. 

**Figure 3:**
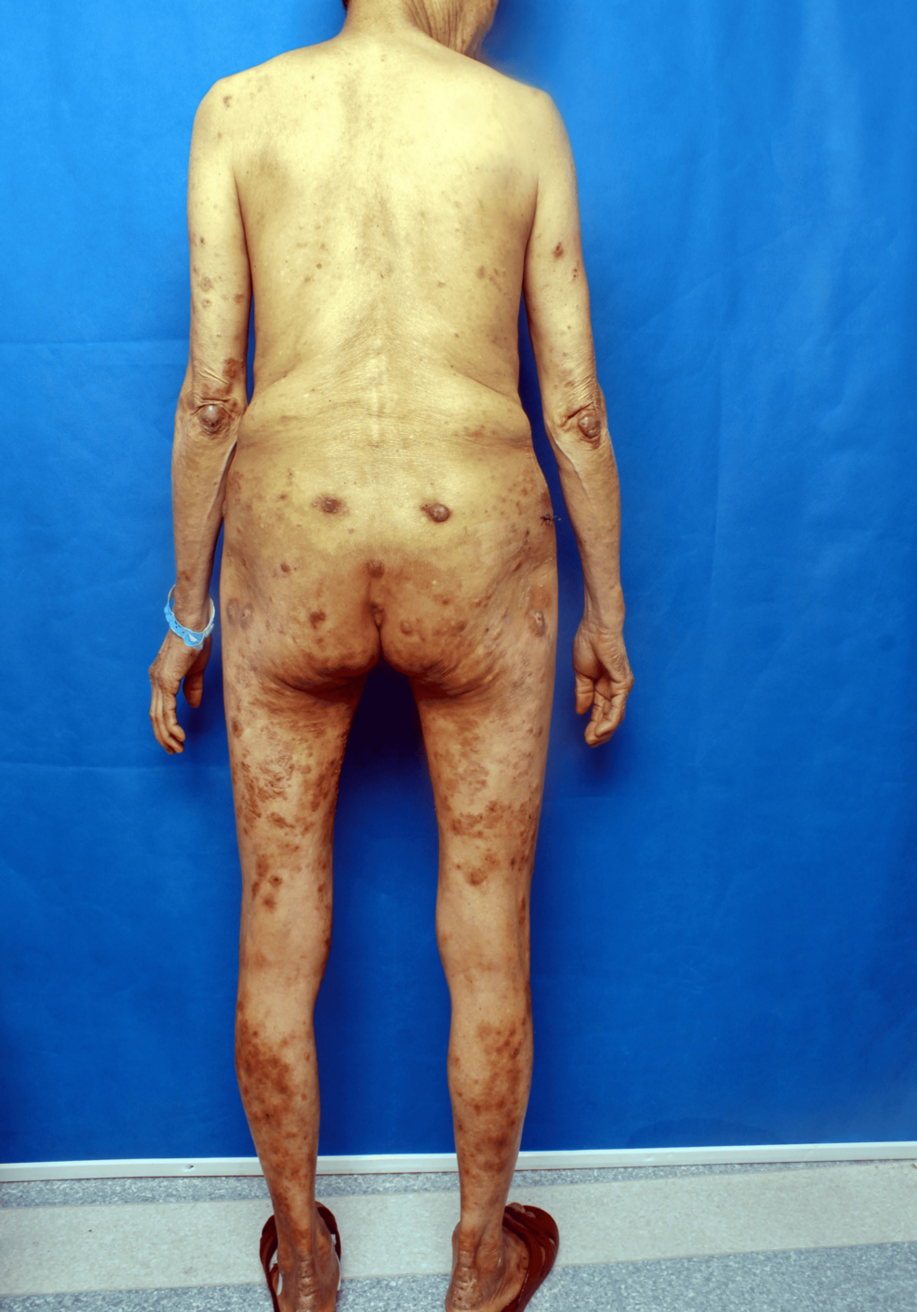
Multiple areas of hyperpigmentation, erythema, and papules on the buttocks and lower limbs

Physical examination revealed extensive hyperpigmentation on the lower extremities and gluteal region, featuring confluent violaceous nodules of varying sizes, erythematous patches, and crusted lesions, particularly in the subgluteal area (Figure 3). Additionally, discrete papules and hyperpigmented macules were noted on the auricular region, trunk, and upper extremities. The rest of the systemic examination was unremarkable.

Laboratory investigations showed elevated serum IgA levels (5.17 g/L) and reactive IgA on serum immunofixation electrophoresis. Genetic testing confirmed positivity for HLA-B13:01. Blood work, including complete blood count, biochemistry, antineutrophil cytoplasmic antibodies (ANCA), C-reactive protein, rheumatoid factor, and tumor markers, returned normal or negative results. Further infectious workup, including blood tests for hepatitis B, hepatitis C, syphilis, HIV, streptococcus, and Epstein-Barr virus, was negative. Tests for autoantigens associated with autoimmune blistering diseases (e.g., BP180, BP230, Dsg1, and Dsg3) were also negative. A CT scan of the lungs revealed chronic inflammation in both lungs. A bone marrow biopsy showed no evidence of plasma cell-related disease.

**Figure 4:**
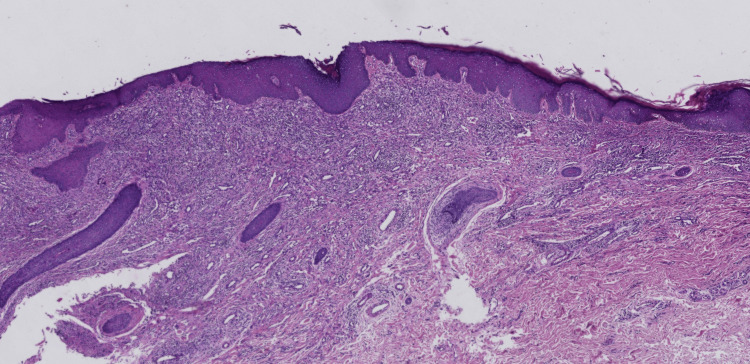
Histopathological examination showing subepidermal blister formation with neutrophilic infiltration around superficial dermal blood vessels (H&E, original magnification ×40)

Histopathological analysis of a biopsy from a buttock nodule revealed subepidermal blister formation, with fibrinoid material and a small number of eosinophils within the blister, accompanied by prominent neutrophilic infiltration around superficial dermal vessels (Figure 4). Direct immunofluorescence microscopy revealed linear deposition of IgM and C3 along the basement membrane zone.

**Figure 5:**
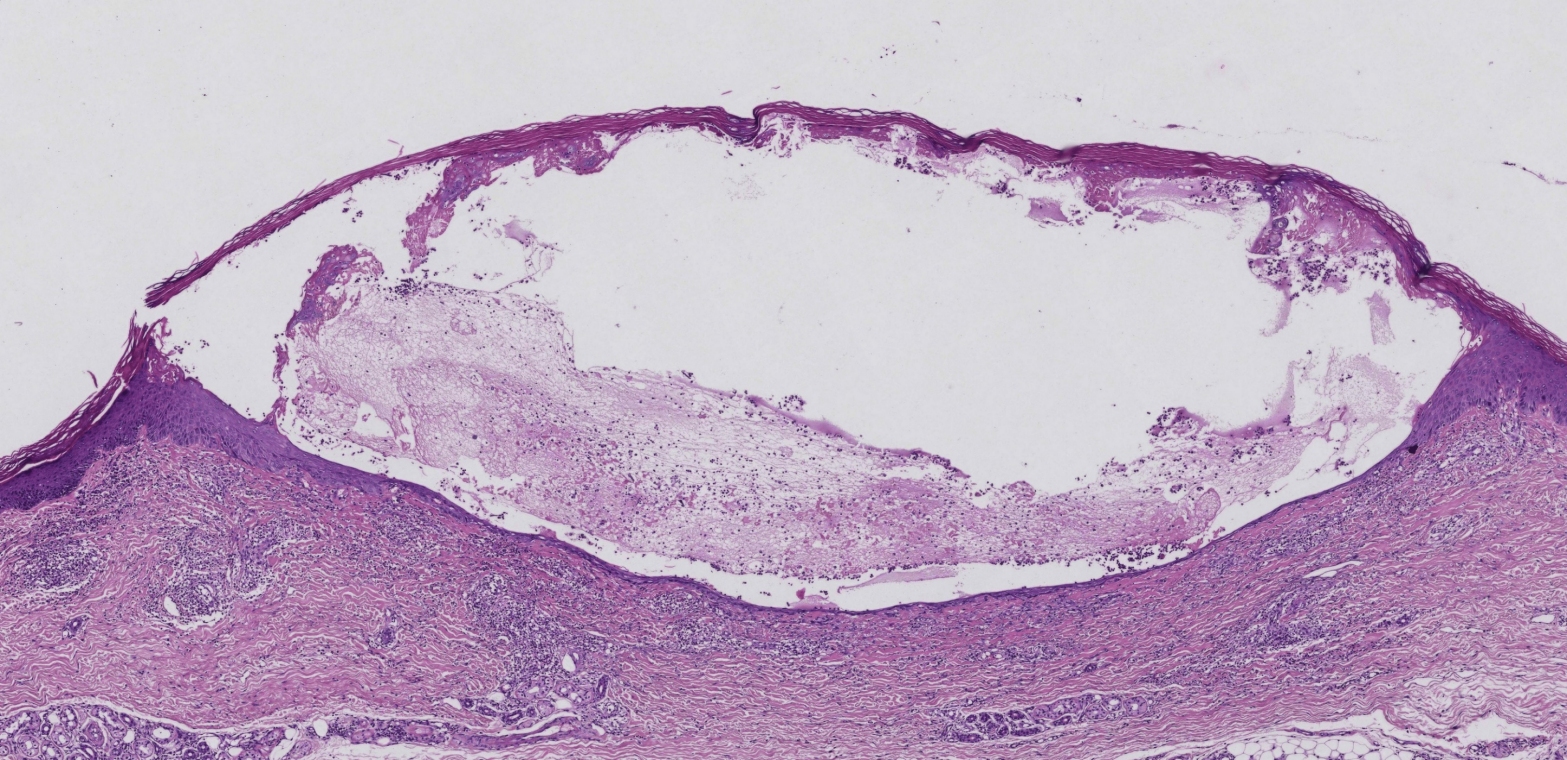
Histopathological examination of the blister lesion revealing a tense intraepidermal blister with moderate neutrophilic infiltration within the epidermis (H&E, original magnification ×40)

**Figure 6:**
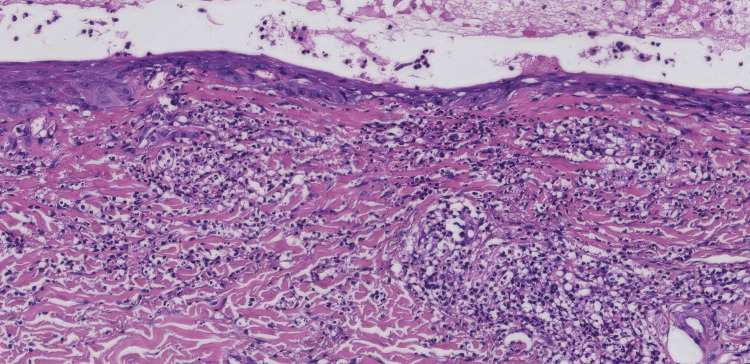
Nuclear debris surrounding the blood vessels (H&E, original magnification ×200)

Furthermore, histopathology of the newly-formed vesicles on the left foot demonstrated intraepidermal tense vesicles, moderate infiltration of lymphocytes, neutrophils, and scattered eosinophils in the superficial dermis, with leukocytoclastic debris surrounding the blood vessels (Figures 5-6). Direct immunofluorescence testing was negative.

Based on the clinical and histopathological findings consistent with leukocytoclastic vasculitis, particularly the subepidermal blister formation, a diagnosis of bullous EED was confirmed. The patient was initiated on a therapeutic regimen consisting of oral prednisone (30 mg daily), thalidomide (50 mg twice daily), intravenous glycyrrhizin complex, antihistamines, and topical corticosteroids. Intradermal injections of triamcinolone (40 mg per injection) were performed on all the nodular skin lesions. After two months of treatment, the patient discontinued all medications on their own, and the rash recurred. The patient resumed oral prednisone (30 mg daily) and thalidomide (25 mg once daily). Subsequently, the prednisone dose was tapered to 20 mg/day. This treatment led to a marked improvement in the patient's clinical condition, with significant regression and flattening of the skin lesions and relief of pruritus. The patient has been followed up for four months without recurrence. The patient continues to be monitored.

## Discussion

EED is a rare chronic dermatosis, typically presenting with firm, purple-red nodules, papules, and plaques on the extensor surfaces of the limbs. First described by Hutchinson in 1888 [4], EED was later recognized as a distinct entity by Radcliffe-Crocker and Williams in 1894 [5]. It most commonly affects adults between the ages of 40 and 60, though cases in children have also been reported. EED shows no gender or racial predilection [6]. It is frequently associated with systemic symptoms such as arthralgia, scleritis, uveitis, marginal ulcerative keratitis, and other ocular lesions. Neuropathy and mucosal ulcers in the oral cavity and esophagus may also occur, with arthralgia being the most prevalent [7]. Due to the broad spectrum of clinical manifestations, diagnosing EED can be challenging. In clinical practice, it must be differentiated from conditions such as granuloma faciale, Sweet syndrome, rheumatoid neutrophilic dermatosis, sarcoidosis, nodular xanthoma, rheumatoid nodules, and annular granuloma. In HIV-infected patients, EED must also be distinguished from Kaposi's sarcoma or bacillary angiomatosis [1]. Histopathological examination in this case revealed subepidermal blister formation with neutrophilic infiltration in the superficial dermis, nuclear debris around blood vessels, consistent with leukocytoclastic vasculitis, in line with the vesicular variant of EED.

The pathogenesis of EED remains poorly understood [8], but it is believed to involve immune complex deposition in small cutaneous vessels, triggering an inflammatory response. This deposition may be associated with chronic antigen exposure or elevated circulating antibody levels, culminating in leukocytoclastic vasculitis. EED is classified as a neutrophilic dermatosis, characterized by the infiltration and activation of neutrophils in the inflamed areas. Endothelial-leukocyte adhesion molecules and cytokines like interleukin-8 (IL-8) and granulocyte-macrophage colony-stimulating factor (GM-CSF) play a significant role in neutrophil activation and exacerbation of the inflammatory process, which may contribute to vesicle formation [9].

Although clinically uncommon, EED may present with vesicular and bullous lesions. This article provides a comprehensive review of the clinical features of bullous EED reported from 2005 to 2025, which are summarized in Table 1. The formation of vesicles is likely due to neutrophil activation and the release of proteolytic enzymes induced by immune complex deposition, which disrupts the basement membrane [9]. Direct immunofluorescence testing in this case showed linear deposits of IgM and C3 along the basement membrane zone, supporting the hypothesis of an immune complex-mediated pathogenesis.

**Table 1:** Bullous erythema elevatum diutinum cases with relevant clinical features (-): Not mentioned in the text

Age (years)	HIV status	IgA levels	Treatment	Outcomes	Author and Year
10	-	-	Topical corticosteroids	Improved	Yuan et al., 2025 [10]
65	Negative	-	Dapsone	Improved	Yalici-Armagan et al., 2022 [11]
49	Negative	Normal	Dapsone	Improved	Ba et al., 2018 [12]
16	Negative	-	Without treatment	Regressed	Ossorio-García et al., 2017 [13]
53	Negative	-	Dapsone	Regressed	Yang et al., 2014 [14]
5	Negative	-	Glycyrrhizin and indomethacin	Improved	Wang et al., 2014 [15]
56	-	Elevated	Glucosidorum tripterygll totorum and nicotinamide	Regressed	Qu et al., 2013 [2]
68	-	-	Dapsone and hydroxychloroquine	Improved	Agusti-Mejias et al., 2011 [16]
35	Positive	-	Dapsone	Improved	Smitha et al., 2011 [17]
9	-	-	Dapsone	Improved	Tomasini et al., 2006 [18]
35	-	-	Dapsone	Improved	Yamamoto et al., 2005 [19]

Some researchers suggest that the flare-ups of EED lesions may correlate with the activity of underlying systemic diseases [20], which include infections, hematologic disorders, autoimmune conditions, and malignancies. Among hematologic disorders, EED is most frequently associated with IgA monoclonal gammopathy, which can evolve into multiple myeloma [21,22]. Therefore, patients with elevated serum IgA should be closely monitored. In this case, the patient exhibited elevated serum IgA levels, which warrant ongoing surveillance as EED can precede hematologic conditions, including multiple myeloma.

While dapsone remains the first-line treatment for EED, other options include corticosteroids, thalidomide, methotrexate, chloroquine, and niacinamide [1]. Surgical excision is considered for advanced fibrotic nodules, as dapsone is less effective for these lesions. The patient’s HLA-B13:01 positivity raised concerns about dapsone hypersensitivity syndrome; therefore, corticosteroids, immunosuppressive agents, and local therapies were used successfully.

## Conclusions

This case underscores the importance of considering bullous EED in the differential diagnosis of chronic vesiculobullous dermatoses. Histopathological evaluation is essential for definitive diagnosis, and regular monitoring for underlying systemic conditions, especially hematologic disorders, is crucial. For patients contraindicated for dapsone therapy, combination therapy with corticosteroids and immunosuppressive agents provides an effective alternative.
